# Salidroside ameliorates abnormalities in electrophysiological indices induced by perfusion of the heart with low-potassium solutions

**DOI:** 10.3389/fcvm.2025.1628940

**Published:** 2025-08-12

**Authors:** Ruoning Yang, Chi Yan, Yifeng Zhou, Wenyuan Li, Gongxin Wang, Huan Li, Fei Lin, Guoliang Hao

**Affiliations:** ^1^ Department of Cardiology, The First Affiliated Hospital, XinXiang Medical University, Xinxiang, Henan, China; ^2^ Henan Engineering Research Center for Clinical Treatment of Coronary Heart Disease, The First Affiliated Hospital of Xinxiang Medical University, Xinxiang, China; ^3^ Henan Joint International Research Laboratory of Cardiovascular Injury and Repair, The First Affiliated Hospital of Xinxiang Medical University, Xinxiang, China; ^4^ Department of Research, Scope Research Institute of Electrophysiology, Kaifeng, Henan, China

**Keywords:** hypokalaemia, salidroside, transcriptomic analysis, cardiac electrophysiology, arrhythmia

## Abstract

Malignant arrhythmias related to hypokalemia are a key risk factor for cardiac arrest, but the specific mechanism remains unclear. In this study, using electrophysiological mapping and transcriptomics techniques, the effects of hypokalemia and paclitaxel (SAL) on isolated rat hearts were investigated. Hypokalemia (3.5–2.0 mmol/L) dose-dependently triggered abnormal arrhythmias and increased the incidence of arrhythmias, while SAL (5 ug/ml) improved this situation. Transcriptomics revealed that hypokalemia upregulated Mt-nd6 and disrupted the inflammatory/immune pathways, while SAL reversed these changes and activated PPAR-related genes. SAL improves the electrophysiological abnormalities caused by hypokalemia by regulating inflammation, immunity and energy metabolism, and has the potential to treat related arrhythmias.

## 1 Introduction

Arrhythmia is one of the most common cardiovascular diseases worldwide and is a leading cause of sudden cardiac death (1). Arrhythmia is an abnormality in heartbeat frequency or rhythm of the caused by obstruction of cardiac activity origin or conduction and is associated with morbidity and mortality in many diseases.

Hypokalaemia is one of the most common electrolyte disorders in clinical practice. According to national and international epidemiological studies, the incidence of hypokalaemia ranges from 2.6–23.2% in hospitalised patients (2), reaching 49.9% in patients with emergency conditions (3), 56% in patients receiving diuretics (4), and 70.4% in patients receiving gastrointestinal surgery (5). There are many drugs used in the clinic that can cause QT interval prolongation, including antidepressants, nonsedating antihistamines, antimicrobials, antipsychotics, antineoplastic drugs, and cardiac drugs (6–10). In a retrospective study, 71% of patients used medications with the potential to prolong the QT interval, and 11% took at least one medication with a known risk of QT interval prolongation (11). When QT interval-prolonging drugs are used by patients with hypokalaemia, the chance of arrhythmia is greatly increased, resulting in more uncertainty regarding their clinical effects.

The normal serum potassium ion concentration ranges from 3.5–5.0 mmol/L, although 3.5–5.5 mmol/L is also commonly used as the standard range in China. Hypokalaemia is defined as a serum potassium concentration less than or equal to 3.5 mmol/L. This threshold is widely recognised, but there is some disagreement on how to grade the severity of hypokalaemia (12). The current criteria for assessing hypokalaemia severity, which are relatively well accepted, are as follows: mild hypokalaemia: 3.0–3.5 mmol/L; moderate hypokalaemia: 2.5–3.0 mmol/L; and severe hypokalaemia: <2.0 mmol/L (13). Previous studies have shown that even mild episodes of hypokalaemia (blood potassium concentration <3.5 mmol/L) are significantly associated with the development of ventricular arrhythmias, with a clear clinical correlation in patients at high risk for ventricular arrhythmias and sudden cardiac death. Moderate hypokalaemia can cause severe arrhythmia in the normal heart, and severe hypokalaemia can induce atrial fibrillation, ventricular fibrillation, tip torsion, ventricular tachycardia and other malignant arrhythmias (14).

In clinical practice, Rhodiola is widely used to prevent altitude sickness (15). Salidroside (SAL) is the dried root and rhizome of Salidroside grandiflora, a member of the Sedum family. SAL is one of the most effective active traditional Chinese medicine ingredients, and recent studies have shown that SAL has anti-hypoxic, antioxidative stress, anti-inflammatory, and glucose-lowering effects (16, 17). Additionally, studies have shown that SAL can reduce inflammatory responses through inhibition of the MAPK signalling pathway, STAT 3 pathway and other inflammatory pathways and reduce the occurrence of ventricular or atrial arrhythmia (18, 19).

Mitochondria play a key role as energy suppliers for the body, and mitochondrial ion channels are key players in regulating mitochondrial function (20). Previous studies have shown that the activation of mitochondrial potassium channels is cardioprotective and that inhibition of these channels can lead to cell death (21). In recent years, studies have revealed a link between electrophysiological and immunological function (22), and mitochondrial ion channels also appear to play a role in the inflammatory response (23); however, information on their role in activating the inflammatory response is still lacking. It is known that hypokalaemia induces cardiac arrhythmia and affects cardiac function, which in turn induces tissue hypoxia and cellular damage. Moreover, hypokalaemia can significantly inhibit or promote the expression of a variety of intra- and extracellular potassium channels, so we speculate that it also plays a role in oxidative stress and inflammation through its effects on mitochondrial potassium channels.

In this study, the isolated hearts of Sprague–Dawley (SD) rats were studied, and cardiac mapping was performed. Hypokalaemia was simulated with a Langendorff perfusion device, and matrix multichannel electrical mapping of isolated rat hearts was performed to exclude the effects of the renin-angiotensin-aldosterone system on the heart and potassium metabolism (24); additionally, the effects of hypokalaemia on the electrophysiological function of the heart were studied. We also used the traditional Chinese medicine ingredient SAL to treat hypokalaemia. Salidroside has been confirmed in previous studies to have anti-tumor and cardiovascular protective effects, the commonly used dose range in *in vitro* experiments is 100 mg/kg (25, 26). In this study, a drug concentration of 5ug/ml was used for cardiac perfusion to confirm its effectiveness and to rule out any obvious toxicity. We also conducted high-throughput transcriptomic analysis to explore the pathways that may be affected by hypokalaemia and SAL to provide a reference for hypokalaemia research, clinical diagnosis and treatment.

## 2 Materials and methods

### 2.1 Experimental animals

Experimental animals: Healthy adult male SD rats (8 weeks old, 300 ± 20 g) were purchased from SPF Biotechnology Co. (Beijing) and Henan Province SCBS Biotechnology Co. All the animal experiments were approved by the Animal Research Ethics Committee of Scope Research Institute and the Ethics Committee of the First Affiliated Hospital of Xinxiang Medical College and were performed in accordance with the Measures for Quality Management of Laboratory Animals.

### 2.2 Experimental drugs and reagents

SAL (catalogue No. IS0020, purity ≥98%) was obtained from Beijing Solarbio Science and Technology Co., Ltd. A 50 mg/ml stock solution of SAL was prepared in KH solution and was stored at −4°C in the dark; the stock solution was used within one week. The relevant experimental equipment, reagent and K-H solution configuration methods are as follows (Supplementary Table S1–S3).

## 3 Experimental methods

### 3.1 Experimental groups

The experimental rats were divided into a control group (*n* = 4), an experimental group (*n* = 8), and a drug administration group (*n* = 4). The rats in the control group were then divided into the control, blank 1, blank 2, blank 3, blank 4, and wash groups, the blank group was used as a negative control for time matching. It was perfused with normal potassium concentration K-H solution simultaneously with the experimental group. The rats in the low-potassium group was divided into the control, 3.5 mM, 3.0 mM, 2.5 mM, 2.0 mM, and wash groups. Moreover, the rats in the drug administration group were divided into the control, 3.5 mM + SAL, 3.0 mM + SAL, 2.5 mM + SAL, 2.0 mM + SAL, wash, and SAL groups. Each individual heart was sequentially perfused with four different concentrations of low-potassium solutions. Unless otherwise specified, the concentration of all drugs was 5 µg/ml, and none of the experimental perfusates were circulated.

### 3.2 Matrix multichannel electrical mapping

A 95% O2 + 5% CO2 gas mixture was perfused, the flow rate was adjusted with a peristaltic pump to the desired value (9–10 ml/min for rats), and the outlet temperature was maintained at 37°C (±0.5°C).

**Figure 1:**
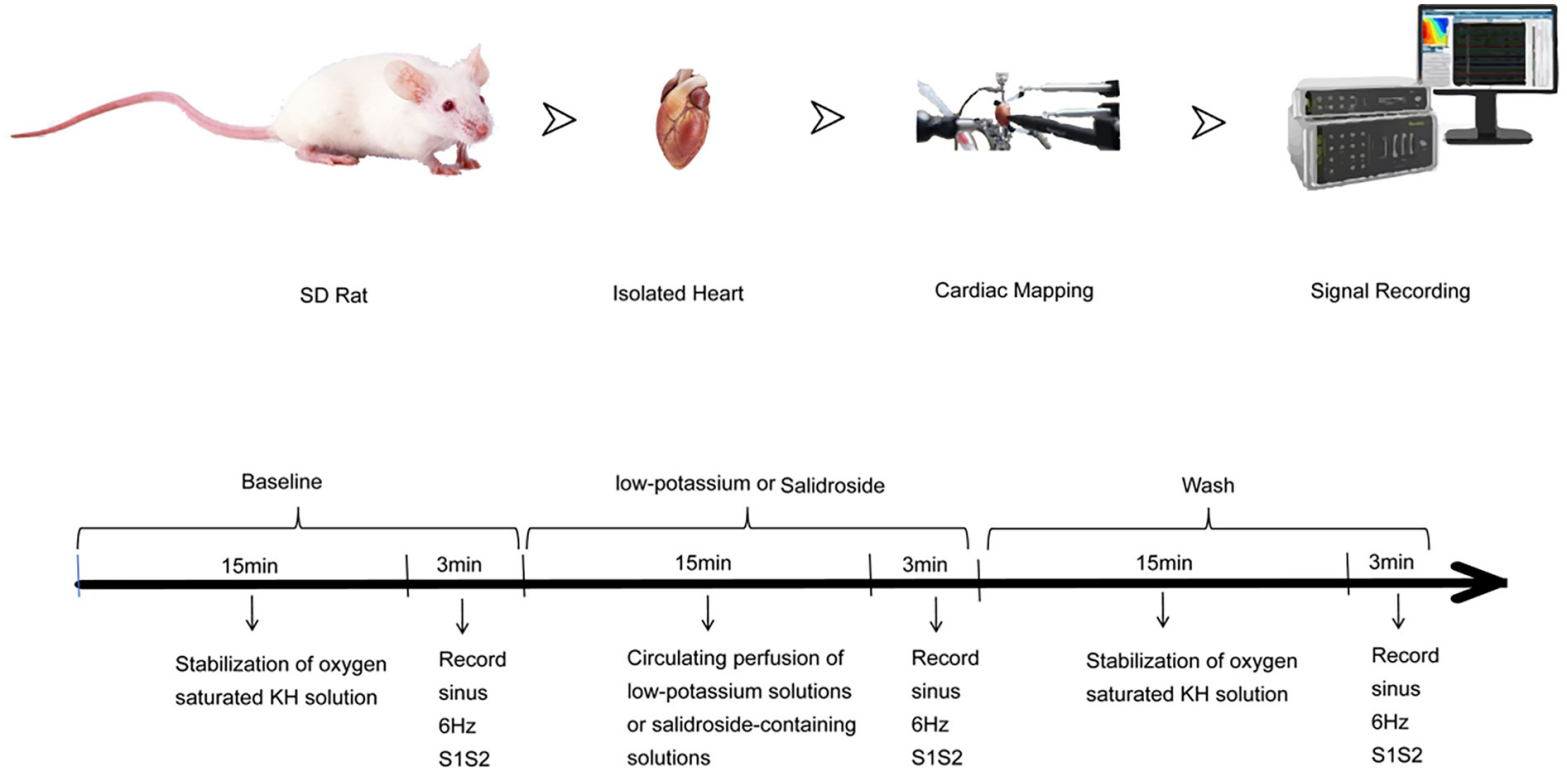
Flow chart of the experiment.

As shown in the Figure 1, the rats were injected intraperitoneally with 3,125 U/kg sodium heparin. The rats were anaesthetised with 0.3 ml of isoflurane gas and then rapidly decapitated and killed. The heart was removed from the chest, the aorta and the surrounding excess tissue were trimmed, and a small amount of perfusate was slowly administered to restore beating and remove residual blood. Then, the heart was quickly transferred to a Langendorff perfusion system, continuously counterirrigated, and reperfused with a small volume of perfusate. The heart was allowed to return to a stable rhythm [heart rate above 260 beats/minute, regular rhythm without premature beats, normal electrocardiogram (ECG) waveform]. After 15 min of stable cardiac perfusion, pen electrodes were attached to the perfusion needle, an ECG electrode was attached to the right atrium and the apical portion of the heart, and a stimulation electrode was attached to the apical portion of the heart. Pen electrodes for channels 1–64 were attached to the left atrium, and those for channels 65–128 were attached to the left ventricle. The surface field potentials of the heart are recorded through electrode arrays, and the conduction time between adjacent electrodes is calculated. The conduction velocity is equal to the electrode spacing divided by the conduction time. The conduction dispersion is the standard deviation of the conduction times in different regions, reflecting the heterogeneity of the electrical activity in different parts of the heart. A constant voltage stimulator was used to deliver stimuli to identify the threshold current, and stimulation at twice the threshold current was delivered to record various indices, such as the ECG and threshold current under 6 Hz stimulation. Then, solutions containing different concentrations of potassium (3.5 mM, 3.0 mM, 2.5 mM, 2.0 mM, and 5.0 mM) were applied for quantitative and qualitative analysis of relationships among various indices under sinus rhythm; indices were recorded at 1, 2, 3, 5, 10, and 15 min. After 15 min, additional stimuli were delivered, the threshold current was remeasured, indices were recorded, and the changes in the heart were observed and recorded. Then, the perfusate was replaced with potassium-containing solutions (3.5 mM, 3.0 mM, 2.5 mM, or 2.0 mM) containing differential concentrations of *Rhodiola rosea* glycosides (5 μg/ml), and the same steps described above were performed.

Software such as Origin Pro 8.0 (Origin Lab) and Adobe Illustrator 10 were used for data analysis and image processing in this study. All experimental data are expressed as the mean ± standard error (mean ± S.E.M.). The significance of differences was analysed using repeated-measures ANOVA.

### 3.3 Transcriptomic analysis

For sequencing, samples were divided into three groups, namely, the blank group, model group, and drug administration group. The potassium concentration administered in the model group and drug administration group was about 2.5 mM (Supplementary Table S4). After the hearts were stabilised for 15–30 min, three different solutions were applied, recordings were made every 10 min, and then the hearts were maintained *in vitro* for 2 h. The samples were frozen in liquid nitrogen for 1 h, placed in a −80°C freezer for storage, and transported on dry ice to BGI Genomtics Co. Ltd. (Shenzhen, China) for transcriptomic analysis.

mRNA isolation was carried out using TRIzol reagent following the manufacturer's instructions. After collecting appropriate tissue samples, a series of steps including grinding, lysis, homogenization, phase separation, RNA precipitation, washing, and resuspension were performed to obtain RNA from myocardial tissues, and mRNA was further enriched with magnetic beads. Subsequently, cDNA (first/second strand) was synthesized, followed by end repair, A-tail addition, adapter ligation, and PCR amplification. The PCR products were heat denatured to single-stranded DNA, which was hybridized with bridge primers to create a single-stranded circular DNA library. After library quality checking, high-throughput mRNA sequencing was conducted. The cyclised DNA generated by rolling circle replication was sequenced on a high-density nanochip using cPAS, and the raw data (SOAPnuke) were filtered to get clean reads. Finally, for the analysis of the mRNA sequencing results, low-quality, junction-contaminated reads, and reads with excessive unknown bases were removed from the raw data to obtain clean reads. The quality of these clean reads was evaluated, and they were compared with the reference genome sequence for differential gene analysis, GO/KEGG enrichment analysis, and other relevant analyses.

## 4 Results

### 4.1 Matrix multichannel electrical mapping results

#### 4.1.1 Effects of solutions containing different low concentrations of potassium on ECG signals of the rat heart

**Figure 2:**
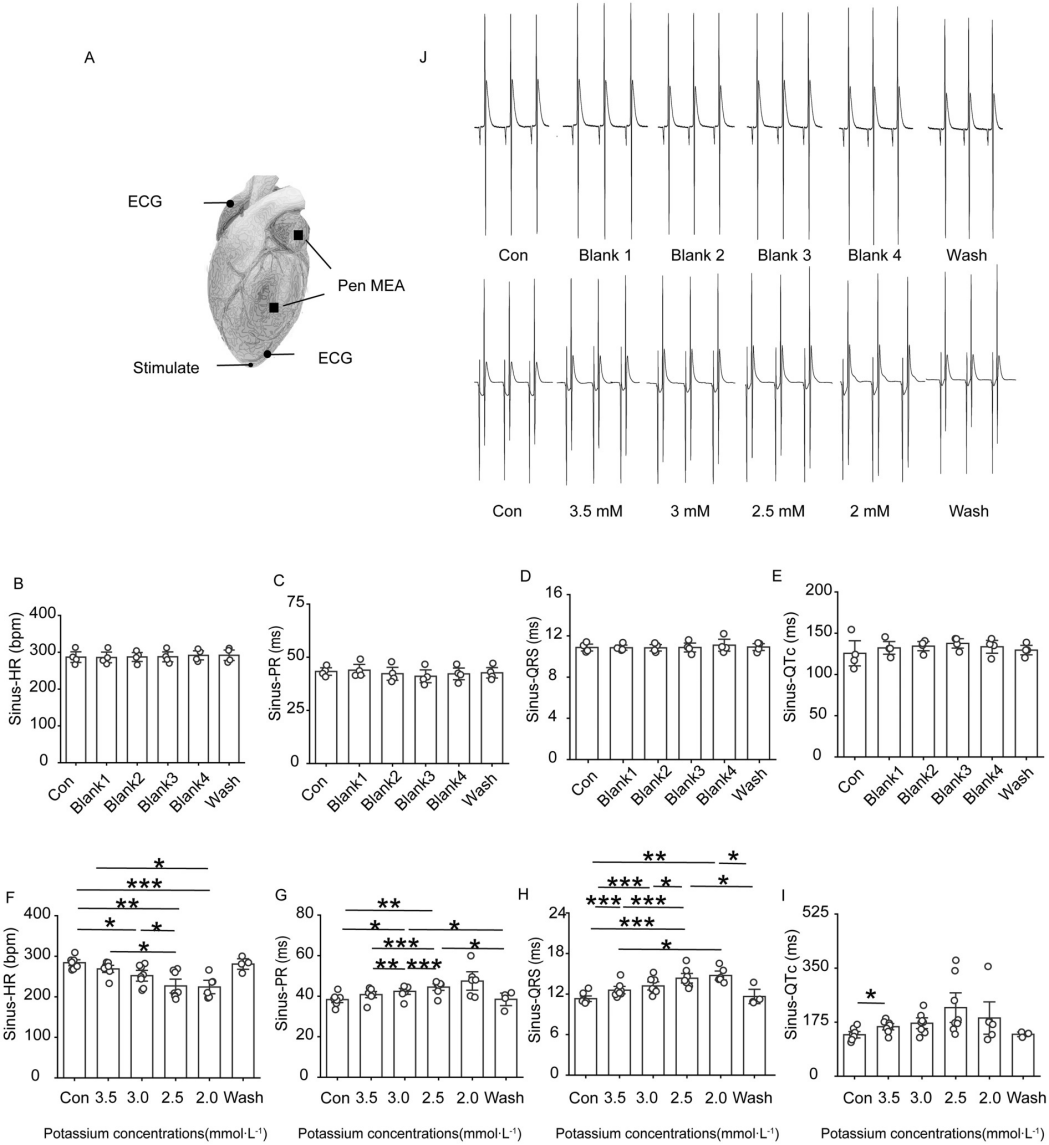
Changes in ECG-related indexes of rat heart. Effect of different concentrations of low potassium solutions (3.5 mM, 3.0 mM, 2.5 mM, 2.0 mM) on ECG recordings in rats (*n* = 8). **(A)** Schematic representation of electrode positions for apical stimulation, multielectrode assay (MEA), and ECG in electrocardiography. **(B–E)** Summary of data representing HR, PR intervals, QRS waves, and QTc intervals in the control group under sinus rhythm from left to right. **(F–I)** From left to right, summarized data on HR, PR intervals, QRS waves, and QT intervals in the low-potassium group under sinus rhythm. **(J)** Representative graphs of ECG waveform changes during perfusion in the control group (Con, Blank 1, Blank 2, Blank 3, Blank 4, and Wash). **(K)** Representative graphs of ECG waveform changes during perfusion in the low-potassium group (Con, 3.5 mM, 3.0 mM, 2.5 mM, 2.0 mM, and Wash). HR, heart rate; PR interval, the interval from the P wave to the R wave; QRS wave, the width of the QRS complex wave; QTc interval, the interval from the Q to the T wave (corrected for heart rate). *p < 0.05, **p < 0.01, ***p < 0.001, ****p < 0.0001.

Rats in the low-potassium group were compared, and the findings revealed that the heart rate significantly decreased when the potassium ion concentration was 2.5 mM and 2.0 mM and that as the potassium ion concentration decreased, the PR interval, QRS interval and QTc interval gradually increased, ultimately yielding statistically significant differences (Figure 2). An extremely significant prolongation of the QT interval was observed on ECG.

#### 4.1.2 Effects of solutions containing different low concentrations of potassium on atrial conduction in rats

**Figure 3:**
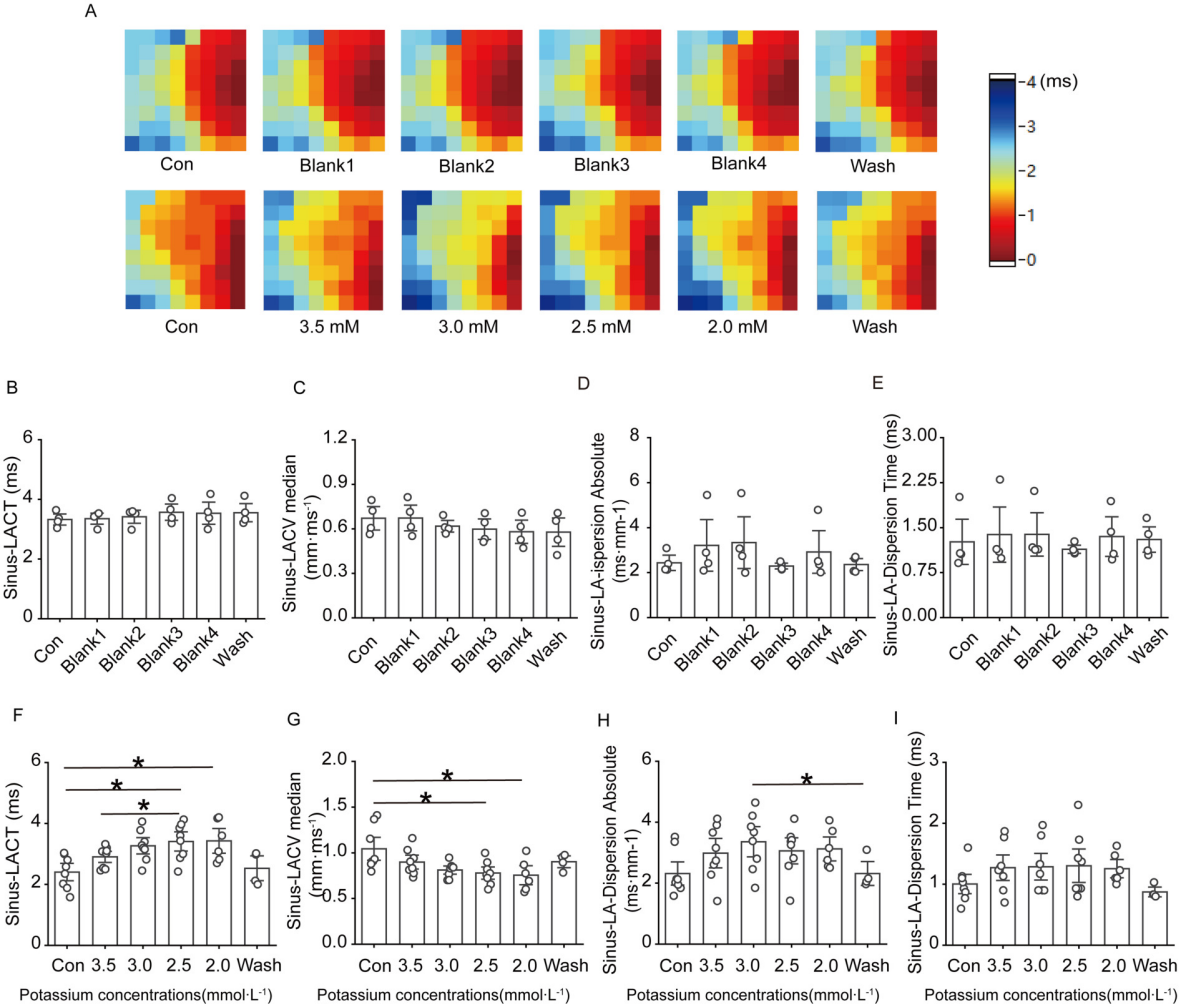
Effect of low potassium perfusion in sinus rhythm on atrial conduction in rats. Sinus-LACT is the left atrial conduction time in sinus rhythm; Sinus-LACV median is the left atrial conduction velocity in sinus rhythm; Sinus-LA-dispersion absolute is the left atrial conduction dispersion in sinus; Sinus-LA-Dispersion Time is the left atrial conduction dispersion in sinus rhythm. **(A)** Representative graphs of the changes in the left atrial conduction thermograms of the control group and the low potassium-treated group during the experiment in sinus rhythm; **(B–E)** Statistical graphs of the left atrial conduction time, conduction velocity, conduction dispersion, and conduction dispersion time in rats treated with K-H solution in sinus rhythm (*n* = 4); **(F–I)** Statistical graphs of left atrial conduction time, statistical plots of conduction velocity, conduction dispersion absolute, and conduction dispersion absolute time in rats treated with low-potassium solution in sinus rhythm (*n* = 8). *p < 0.05, **p < 0.01, ***p < 0.001.

Figure 3A–E shows that, under sinus rhythm, conduction at the electrode placement site on the heart surface in the control group basically remained stable throughout the experimental process, with no obvious changes; in the low-potassium group, as the potassium ion concentration in the solution decreased, the conduction velocity of the left atrium slowed, and the direction of conduction at the electrode placement site moved upwards from downwards and to the left. In the low-potassium group, under sinus rhythm, there was a statistically significant decrease in atrial conduction at ion concentrations of 2.5 mM and 2.0 mM (Figures 3F,G), and atrial dispersion gradually decreased with decreasing potassium ion concentration, exhibiting a statistically significant difference (Figures 3H,I).

#### 4.1.3 Effects of solutions containing different low concentrations of potassium on ventricular conduction in rats

**Figure 4:**
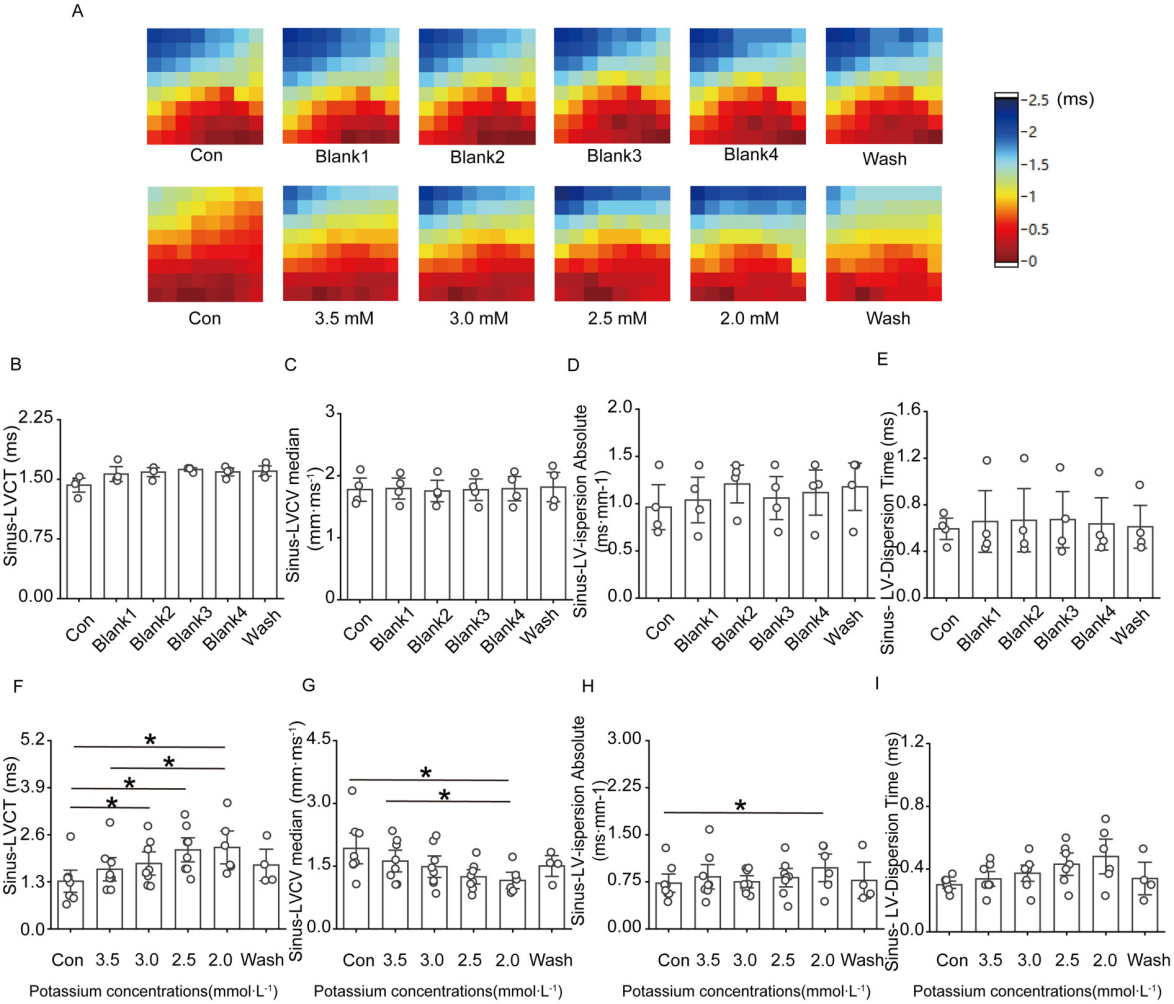
Effect of low potassium perfusion on ventricular conduction in isolated rats in sinus rhythm. Sinus-LVCT is the left ventricular conduction time in sinus rhythm; Sinus-LVCV median is the left ventricular conduction velocity in sinus rhythm; Sinus-LV-dispersion absolute is the left ventricular conduction dispersion in sinus; Sinus-LV-Dispersion Time is the left ventricular conduction dispersion time in sinus rhythm. **(A)** Representative graphs of the left ventricular conduction thermogram changes in the control group and low-potassium-treated groups during the experiment in sinus rhythm; **(B–E)** Statistical graphs of LV conduction time, conduction velocity, conduction dispersion, conduction dispersion time in rats treated with K-H solution in sinus rhythm (*n* = 4); **(F–I)** Statistical graphs of LV conduction time, conduction velocity, conduction dispersion, and conduction dispersion time in rats treated with graded low-potassium solution in sinus rhythm (*n* = 8). *p < 0.05, **p < 0.01, ***p < 0.001.

Figure 4A–E shows that ventricular conduction in the control group basically remained stable as the perfusion time decreased; in the low-potassium group, as the concentration of potassium ions in the solution decreased, the conduction time at the electrode placement site on the surface of the rat heart significantly increased, and the conduction speed gradually slowed; both the conduction time and the conduction speed were significantly different in the presence of 2.5 mM and 2.0 mM potassium (Figures 4F,G). Under sinus rhythm, compared with the control treatment, the solution containing 2.0 mM potassium significantly increased the left ventricular conduction dispersion in rats.

#### 4.1.4 Effects of solutions containing different low concentrations of potassium on ventricular conduction under 6 Hz stimulation in rats

**Figure 5:**
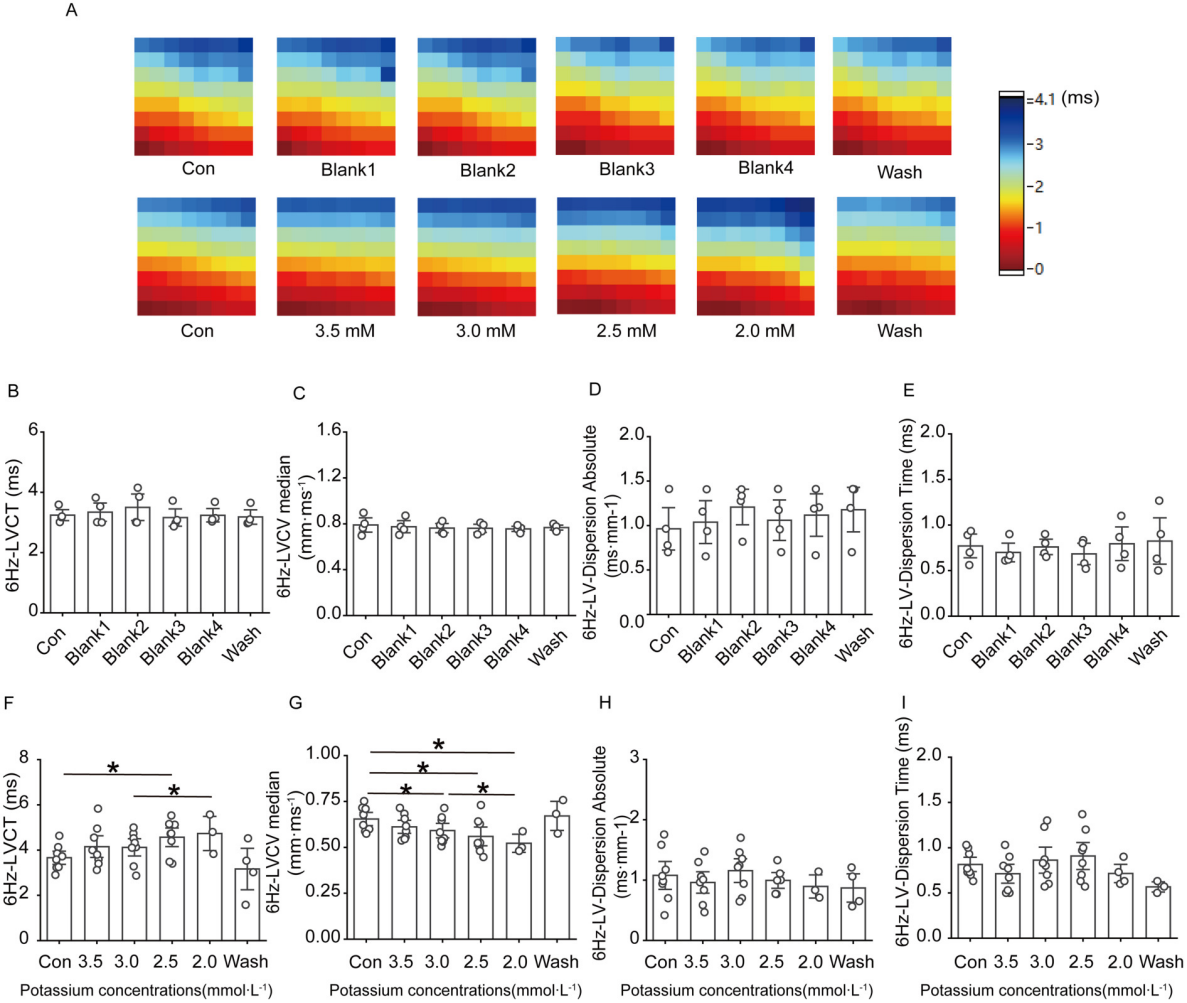
Effect of low potassium perfusion on rat left ventricular conduction under 6 Hz stimulation. 6 Hz-LVCT is the left ventricular conduction time under 6 Hz stimulation; 6 Hz-LVCV median is the left ventricular conduction velocity under 6 Hz stimulation; 6 Hz-LV-dispersion absolute is the left ventricular conduction dispersion under 6 Hz stimulation; 6 Hz-LV-Dispersion Time is the left ventricular conduction dispersion time under 6 Hz stimulation. **(A)** Representative graphs of the left ventricular conduction thermogram changes in control groups and low-potassium-treated groups during the experiment under 6 Hz stimulation; **(B–E)** Statistical graphs of the left ventricular conduction time, conduction velocity, conduction dispersion, and conduction dispersion time in rats treated with K-H solution under 6 Hz stimulation (*n* = 4); **(F–I)** Statistical graphs of the left ventricular conduction time in rats treated with gradient low potassium solution under 6 Hz stimulation (*n* = 8). *p < 0.05, **p < 0.01, ***p < 0.001.

Figure 5A–E shows that ventricular conduction in the control group basically remained stable throughout the experiment; in the low potassium-treated group, as the potassium ion concentration in the solution decreased, the conduction time at the electrode placement site on the surface of the rat heart significantly increased, and the conduction speed gradually decreased (Figures 5F–I).

#### 4.1.5 Effects of solutions containing different low concentrations of potassium on the effective refractory period (ERP), threshold current, and atrioventricular delay (AVD) in rats

**Figure 6:**
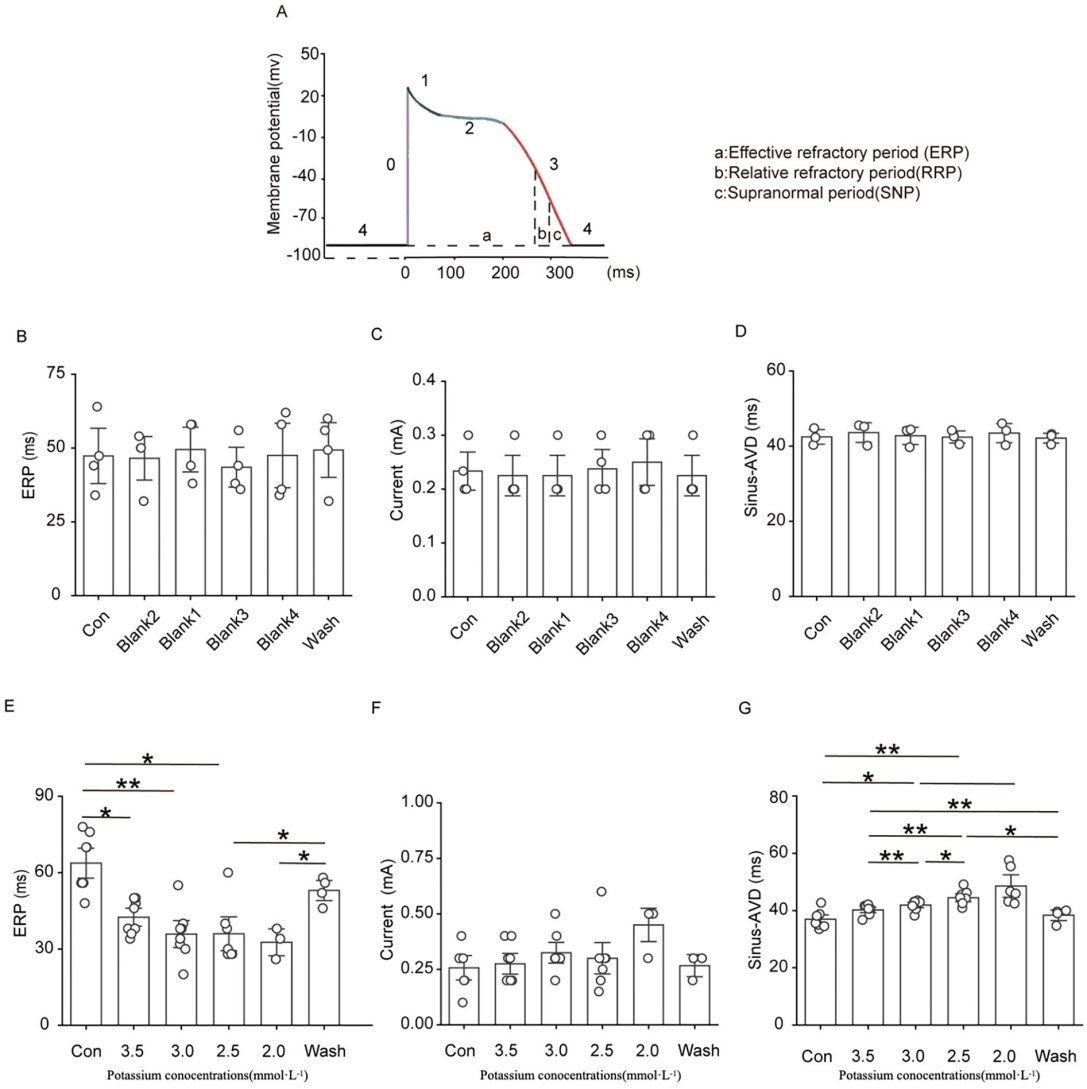
Effect of hypokalemia on the effective refractory period, threshold current, and atrioventricular block in rats under sinus rhythm.

The ERP, threshold current and AVD were relatively stable in the blank group. The threshold current of the isolated rat heart did not change significantly at a potassium ion concentration of 3.5 mM, 3.0 mM, or 2.5 mM, and there was a clear trend of an increasing threshold current when the potassium ion concentration was reduced to 2.0 mM, although the difference was not significantly different. As shown in Figure 6, solutions containing low concentrations of potassium significantly shortened the ventricular ERP and prolonged the repolarisation duration. The shortening of the ERP was most pronounced in 3.5 mM potassium group compared with the control group, and the ERP was reduced to less than 50% of the initial value in the presence of 2.0 mM potassium. Solutions containing 3.0 mM, 2.5 mM, and 2.0 potassium significantly prolonged the AVD under sinus rhythm, and this change was rapidly reversed after elution with a solution containing a normal concentration of potassium ions.

#### 4.1.6 Incidence of cardiac arrhythmia in rats

Various types of arrhythmias were observed in isolated rat hearts perfused with solutions containing different concentrations of potassium ions, and we analysed the incidence of arrhythmia in the rat heart induced by solutions containing various low concentrations of potassium (Table 1). The results revealed that the frequency of atrial parasystole and ventricular parasystole was the highest, followed by atrial fibrillation. A total of 4 rats exhibited direct ventricular fibrillation, and 1 rat exhibited atrial fibrillation followed by ventricular fibrillation during the experiment.

**Table 1:** Statistics of types of arrhythmias occurring at different potassium ion concentrations.

n	3.5 mmol·L^−1^	3.0 mmol·L^−1^	2.5 mmol·L^−1^	2.0 mmol·L^−1^
1	/	/	PAB	PVB、Bradycardia、SCA、VF
2	PAB	/	/	VF
3	PAB	PAB	PAB	Bradycardia、VF、SCA
4	/	PAB	VT、PVB、VF	/
5	/	VT	VF	/
6	/	PVB	PVB、PAB	PVB、PAB、AVB、AF
7	/	/	Sinus Arrhythmia	/
8	/	/	PAB、PVB	PAB

/, No arrhythmia occurred; PAB, Premature Atrial Beats; PVB, Premature Ventricular Beats; VT, Ventricular Tachycardia; VF, Ventricular Fibrillation; SCA, Sudden Cardiac Arrest; AF, Atrial Flutter; AVB, Atrioventricular Block.

#### 4.1.7 Effects of SAL on cardiac ECG signals in rat hearts perfused with solutions containing low concentrations of potassium

**Figure 7:**
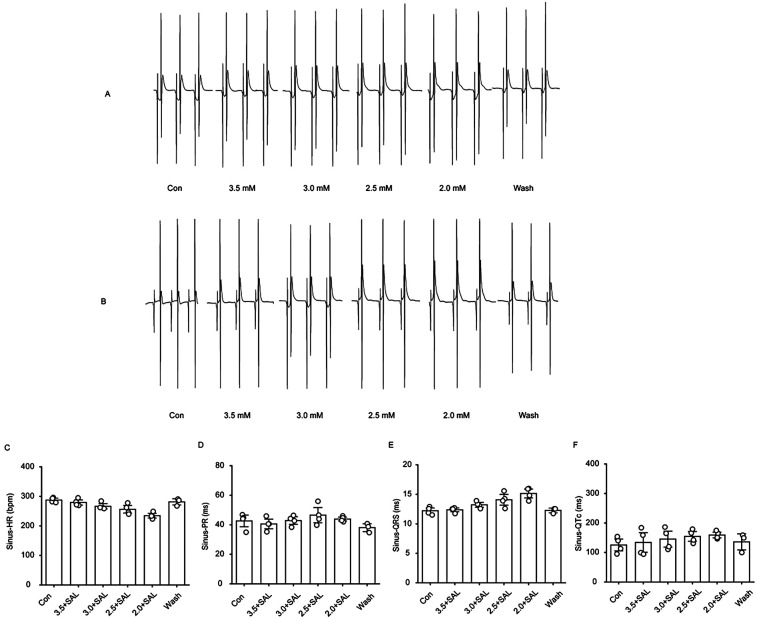
Changes of ECG-related indexes in the heart of hypokalemic rats treated with salidroside. **(A)** Representative graphs of ECG waveform changes in the gradient low-potassium-treated group during the experimental process. **(B)** Representative graphs of ECG waveform changes in the gradient low-potassium-treated group with the addition of Salidroside during the experimental process. **(C)** Statistical graphs of the effects of heart rate, PR interval, QRS interval, and QTc interval in rats in the Salidroside-treated group (*n* = 4). *P < 0.05, **P < 0.01, ***P < 0.001.

Figure 7 shows that after rat hearts were perfused with low-potassium solution containing 5 µg/ml SAL, trends towards a decrease in heart rate, prolongation of the PR interval, prolongation of the QRS interval, and prolongation of the QT interval were observed; however, only the QRS interval exhibited difference in the presence of 2.0 mM potassium, although the difference was not statistically significant between groups.

#### 4.1.8 Effects of SAL on cardiac conduction in rats

**Figure 8:**
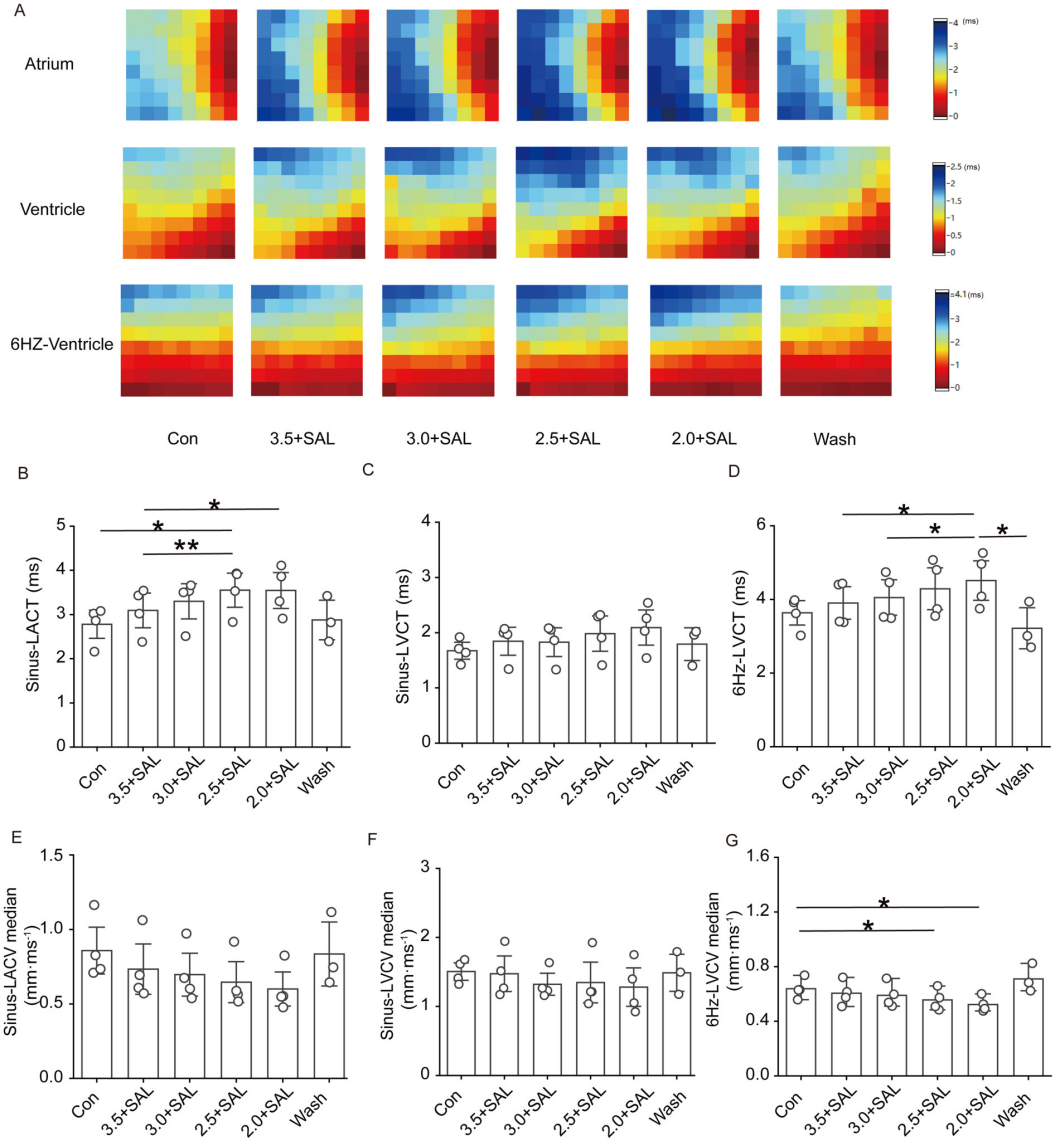
Effect of Salidroside on isolated cardiac conduction in rats of the low potassium treatment group. Sinus-LACT is left atrial conduction time in sinus rhythm; Sinus-LACV median is left atrial conduction velocity in sinus rhythm; Sinus-LVCT is left ventricular conduction time in sinus rhythm; Sinus-LVCV median is left ventricular conduction velocity in sinus rhythm; 6 Hz -LVCT is left ventricular conduction time in 6 Hz stimulation; 6 Hz -LVCV median is left ventricular conduction velocity in 6 Hz stimulation; 6 Hz -LVCV median is left ventricular conduction velocity under 6 Hz stimulation; **(A)** Representative graphs of the changes in the left atrial and left ventricular conduction thermograms of the Salidroside-treated groups during the experimental process under sinus rhythm and 6 Hz stimulation; **(B and C)** Statistical graphs of the left atrial and left ventricular conduction times of the Salidroside-treated groups in sinus rhythm; **(D)** Statistical graphs of left ventricular conduction time in the Salidroside-treated groups under 6 Hz stimulation; **(E and F)** statistical graphs of left atrial and left ventricular conduction velocities in the Salidroside-treated groups under sinus rhythm; **(G)** statistical graphs of left ventricular conduction velocitiesin the Salidroside-treated groups under 6 Hz stimulation, *n* = 4. *p < 0.05, **p < 0.01, ***p < 0.001.

Figure 8 shows that after the administration of low-potassium solution containing 5 µg/ml salidroside, a trend towards decreases in the atrial and ventricular conduction velocities and prolongation of the conduction time were observed under sinus rhythm and under 6 Hz stimulation. In the presence of SAL, the solutions containing the different low concentrations of potassium had no significant effect.

#### 4.1.9 Effects of SAL on conduction dispersion in the rat heart

**Figure 9:**
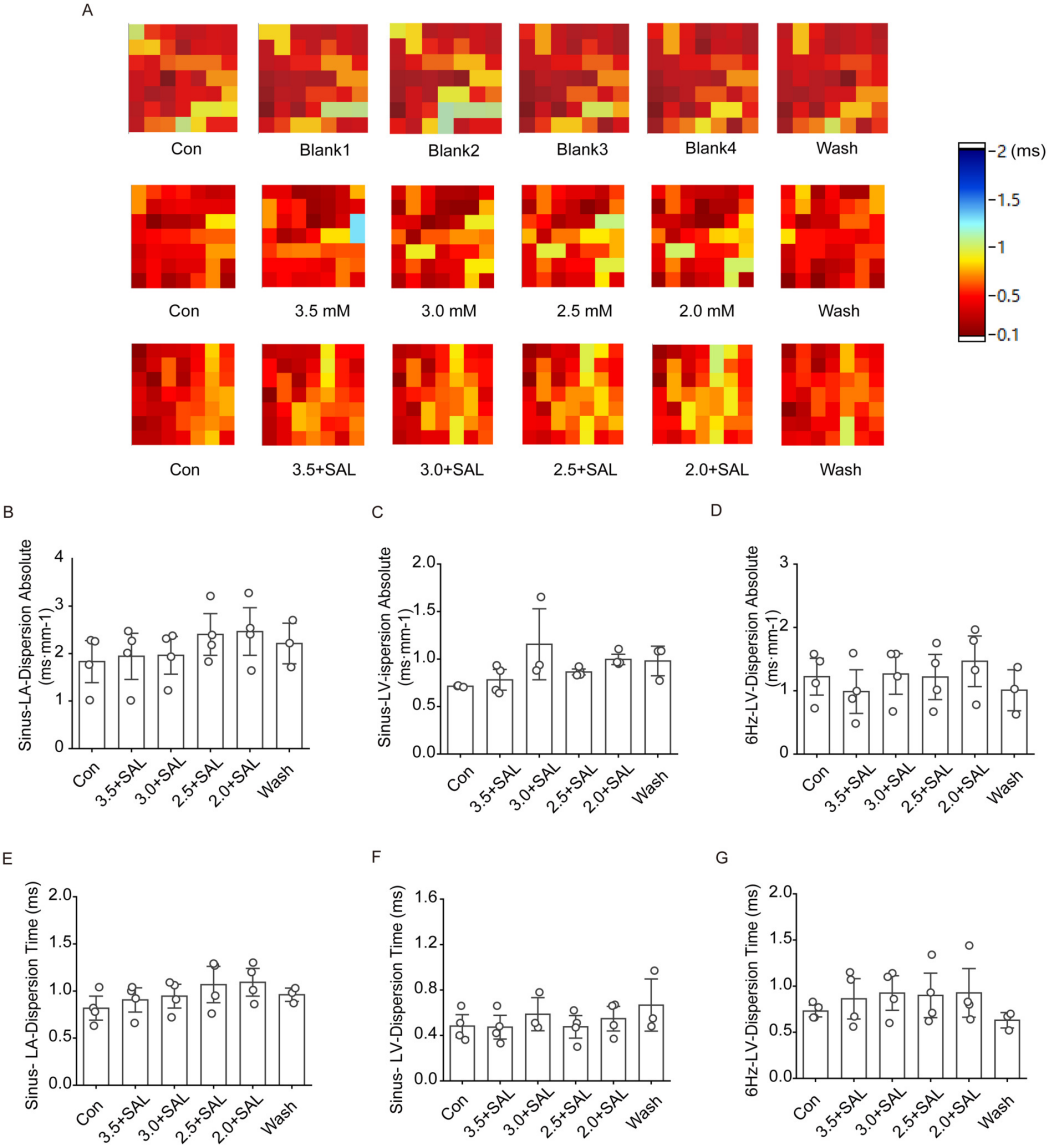
Effect of salidroside on conduction dispersion in isolated hearts of rats in the low potassium-treated group. Sinus-LA-dispersion absolute is left atrial conduction dispersion in sinus rhythm; Sinus-LA-Dispersion Time is left atrial conduction dispersion time in sinus rhythm. Sinus-LV-dispersion absolute is left ventricular conduction dispersion in sinus; Sinus-LV- Dispersion Time is left ventricular conduction dispersion time under sinus rhythm. 6 Hz-LV-dispersion absolute is left ventricular conduction dispersion under 6 Hz stimulation; 6 Hz-LV-Dispersion Time is left ventricular conduction dispersion time under 6 Hz stimulation. **(A)** Thermograms of left atrial conduction dispersion in control, low potassium-treated, and Salidroside-treated groups under sinus rhythm; **(B and C)** Statistical graphs of left atrial and left ventricular conduction dispersion in Salidroside-treated groups under sinus rhythm (*n* = 4); **(D)** Statistical graphs of left ventricular conduction dispersion in Salidroside-treated groups under 6 Hz stimulation (*n* = 4); **(E and F)** Statistical graphs of left atrial and left ventricular conduction dispersion time in Salidroside-treated groups under sinus rhythm (*n* = 4); **(D)** Statistical graphs of left atrial and left ventricular conduction dispersion time in Salidroside-treated groups under 6 Hz stimulation (*n* = 4); **(G)** Statistical graph of left ventricular conduction dispersion time in rats of Salidroside-treated group under 6 Hz stimulation (*n* = 4). *p < 0.05, **p < 0.01, ***p < 0.001.

Figure 9 shows that after the administration of low-potassium solutions containing 5 µg/ml SAL to the rats, the trend towards a gradual increase in atrial and ventricular conduction dispersion was significantly alleviated, but there was no statistically significant increase in atrial or ventricular dispersion at any potassium concentration under sinus rhythm.

#### 4.1.10 Effects of SAL on the ERP, threshold current, and AVD in rats

**Figure 10:**
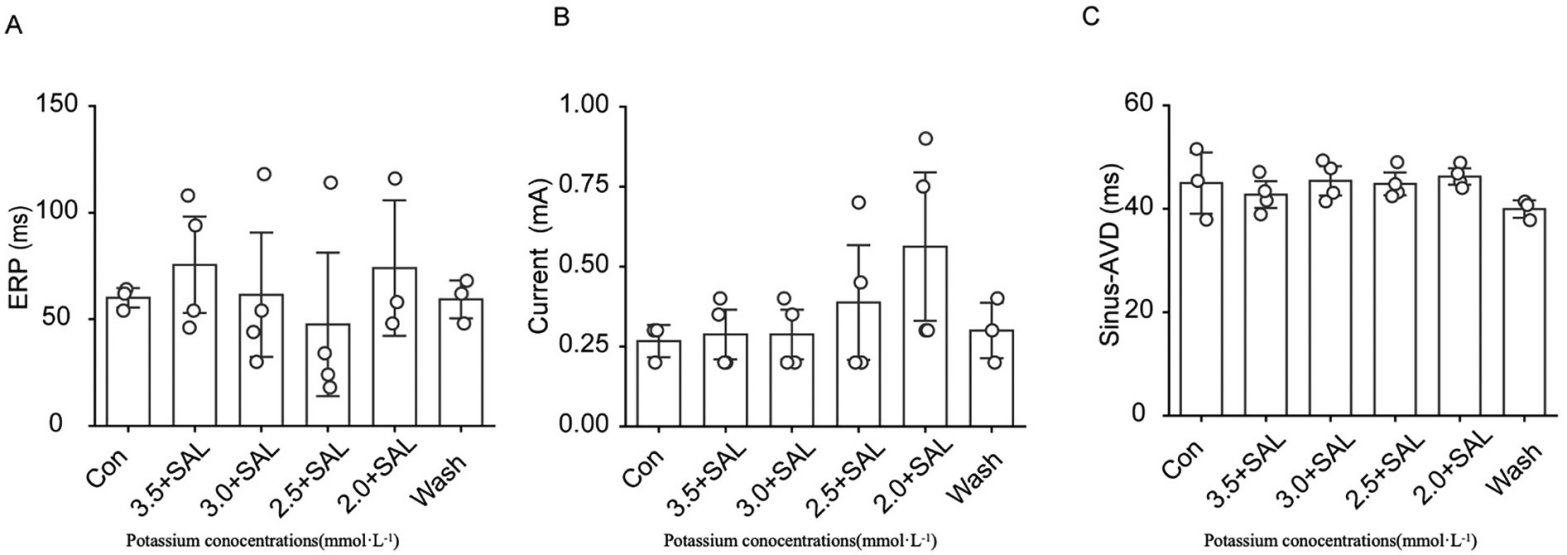
Effects of Salidroside treatment group on ERP, threshold current and AVD in isolated rat heart. Current is the ventricular threshold current. sinus-AVD is the atrioventricular delay in sinus rhythm. **(A)** Statistical graph of cardiac ERP in Salidroside-treated group; **(B)** Statistical graph of cardiac threshold current in Salidroside-treated group; **(C)** Statistical graph of AVD in Salidroside-treated group (*n* = 4). *P < 0.05, **P < 0.01, ***P < 0.001.

After the addition of 5 µg/ml SAL to low-potassium solution, the decrease in the ERP caused by low potassium was significantly attenuated, and the low potassium ion concentration significantly affected the AVD under sinus rhythm (Figure 10). In all of the low-potassium 3.0 mM, 2.5 mM, and 2.0 mM) groups, the AVD was significantly increased under sinus rhythm, while SAL, stabilised the AVD under sinus rhythm. However the trend towards prolongation of the threshold current persisted even in the presence of SAL.

#### 4.1.11 Intergroup comparison

**Figure 11:**
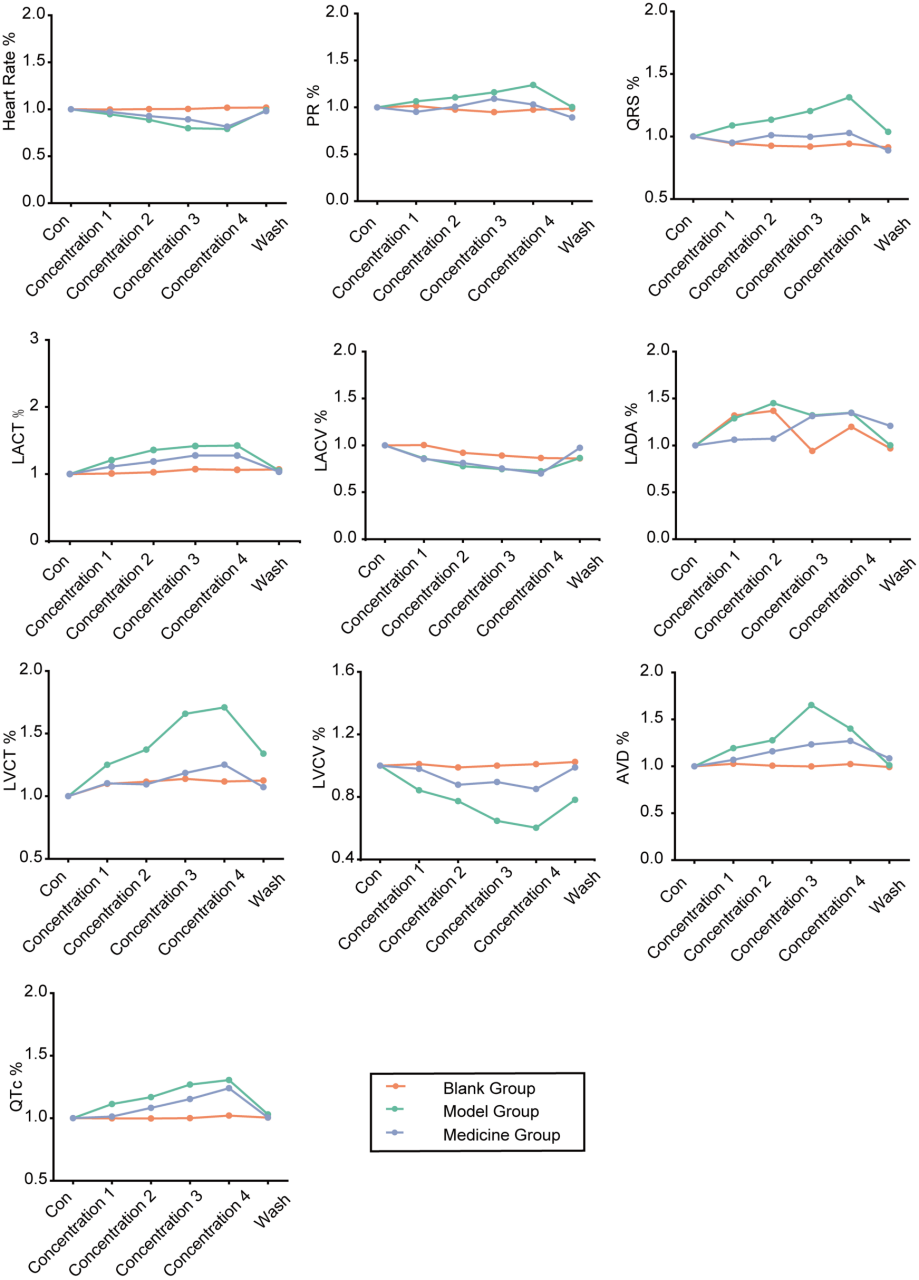
Line graphs of the degree of change in the control, low potassium-treated and salidroside-treated groups (initial state of each rat was standardized to 1).

The degrees of change in the control group, treatment group and drug administration group were calculated, a line graph was constructed, and the differences among groups were statistically analysed (Figure 11). We observed that SAL could alleviate hypokalaemia in the presence of each concentration of potassium, as indicated by normalization of the heart rate, PR interval, QRS interval, and QT interval. The line graph in Figure 11 shows that under sinus rhythm, SAL ameliorated hypokalaemia to some degree in all of the low-potassium solutions, but the differences were not statistically significant. Additionally, there was a statistically significant difference in atrial conduction dispersion between the 3.0 mM potassium group and the SAL-treated group, indicating improved function.

### 4.2 Transcriptomic analysis

**Figure 12:**
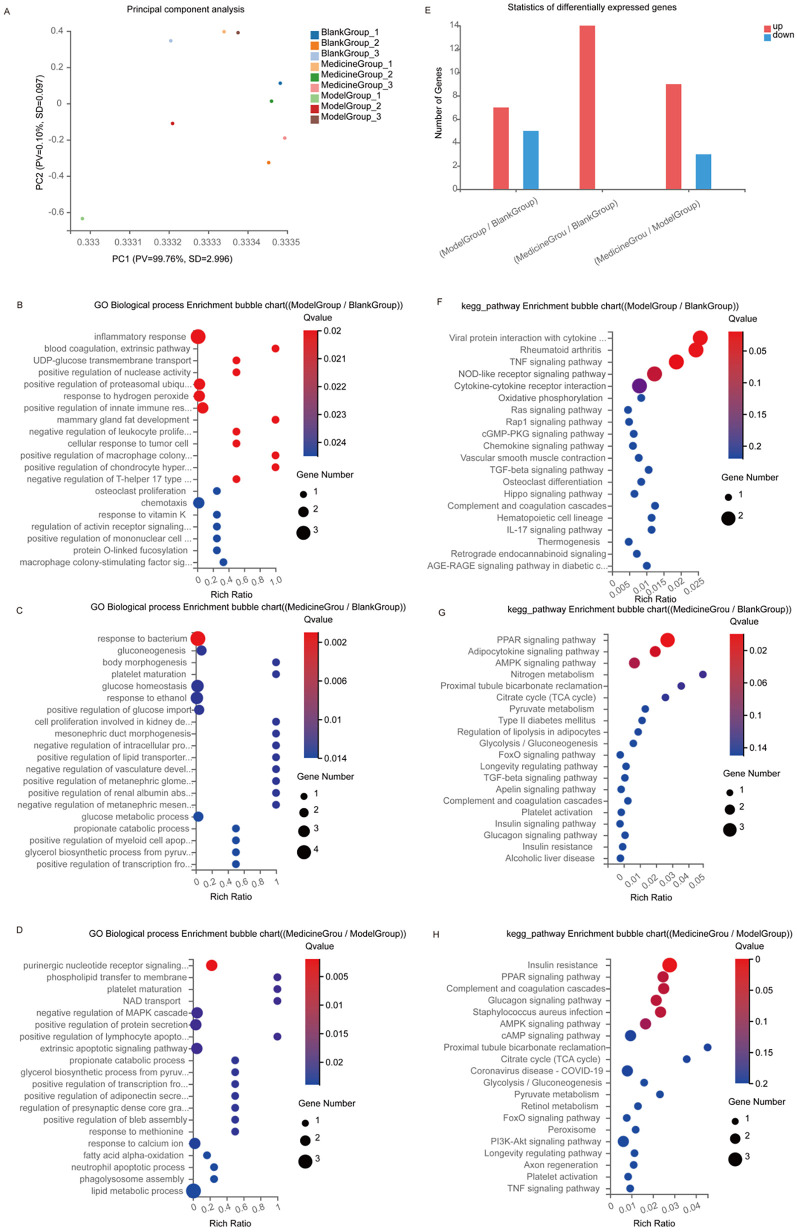
Transcriptomics sequencing results of control, model and administered groups. **(A)** PCA plot; **(B–G)** Top 20 GO biological process enrichment, the vertical axis indicates GO items, and the horizontal axis indicates Rich factors; **(E)** differentially expressed genes; **(F–H)** Top 20 KEGG pathway enrichment, the vertical axis indicates KEGG pathway names, and the horizontal axis indicates Rich factors. The size of the dots indicates the number of genes in that GO/KEGG item, and the color of the dots corresponds to different *p*-value ranges. Blank Group: control group; Model Group: low-potassium-treated group; Medicine Group: Salidroside (5ug/ml) + low-potassium-treated group.

We analysed differentially expressed genes in the hearts of SD rats in the control, low-potassium, and SAL-treated groups. There were large intergroup differences according to PCA (Figure 12A). Among the three groups included in Venn analysis, 15,642 overlapping genes and 322, 371 and 380 unique genes were identified. There were 12 differentially expressed genes, including seven upregulated genes and five downregulated genes, in the low-potassium group compared with the control group, suggesting that a low concentration of potassium can lead to changes in gene expression, including significant upregulation of mitochondria-encoded NADH dehydrogenase 6 (Mt-nd6). There were 14 upregulated genes in the SAL-treated group compared with the control group and nine upregulated genes and three downregulated genes in the SAL-treated group compared with the low-potassium group (Figure 12E). These results suggest that SAL regulates gene expression in low-potassium-treated isolated rat hearts. GO and KEGG analyses were used to further explore the functions of the differentially expressed genes. GO enrichment analysis revealed that low-potassium treatment triggered inflammatory responses but to a minimal degree, considering that this study revealed only a correlative relationship. In addition, low-potassium solutions altered the expression of genes enriched in immune inflammatory processes such as positive regulation of the immune response, negative regulation of leukocyte proliferation, and the macrophage colony-stimulating factor signalling pathway, although these changes were abated after SAL treatment (Figures 12B–D). KEGG pathway enrichment analysis revealed that the genes CSf 1 and Ccl 12, which were significantly downregulated in the low-potassium group compared with the control group, are involved in the MAPK signalling pathway and cell‒cytokine interactions. The expression of both Adipoq and plin 1, which are involved in the PPAR signalling pathway, was upregulated after SAL treatment (Figures 12F–H).

## 5 Discussion

Arrhythmia is a cardiovascular disease characterised with high morbidity rates and high risks, and malignant arrhythmia due to hypokalaemia is one of the most important factors associated with increased clinical mortality (27). In the 1960s, it was reported in the literature that hypokalaemia can cause related ECG abnormalities. In the 1990s, with the development of patch clamp technology, ion channels in cardiomyocytes were discovered, and more than 10 types of potassium ion channels in cardiomyocyte have been identified. Potassium plays an important role in all four phases of the cardiac action potential except phase 0, and it can contribute to the occurrence of ventricular arrhythmia through a variety of electrophysiological mechanisms, including pacing abnormalities, conduction slowdown, repolarisation prolongation, and so on (28).

Our preliminary pre-experiment indicated that during the 5 to 10 min of perfusion, the electrophysiological abnormalities (such as prolonged QT interval and slowed conduction) had not yet stabilized; at 15 min, the changes in various indicators reached a stable state and were reproducible; after 20 min, irreversible damage such as myocardial edema would occur. Based on this, 15 min was selected as the perfusion duration to balance the stability of electrophysiological abnormalities and the activity of myocardial cells. The results of the present study revealed that the risk of malignant arrhythmia in isolated rat hearts was low in the presence of mild hypokalaemia (3.5 mM, 3.0 mM) and that moderate to severe hypokalaemia (2.5 mM, 2.0 mM) was associated with a significantly increased risk of arrhythmia. The heart rate of isolated rat hearts gradually decreased during perfusion with low-potassium solutions, which is consistent with the findings of previous studies. Further studies revealed that the heart rate of the rats was significantly reduced in the mild to moderate hypokalaemia (3.5 mM, 3.0 mM potassium) groups, and the reduction in heart rate was more severe but tended to be slower in the severe hypokalaemia (2.5 mM, 2.0 mM potassium) groups. Additional findings were revealed by a detailed analysis of different ECG waveforms. In addition to the prolongation of the QT interval described in previous literature, the prolongation of the PR interval and QRS interval was also observed in our study, indicating that the slowing of the heart rate induced by a low concentration of potassium occurs throughout the whole cardiac action potential; however, the degree of prolongation was different in each stage, with the prolongation of the QT interval being the greatest. The QT interval induced by low potassium may be related to the rapid delayed rectifier potassium current (I_kr_) and the late sodium current (I_na_). The weakening of Ikr will cause a slower repolarization rate, while the enhancement of Ina will prolong the duration of the action potential. The prolongation of QRS and QT intervals could be reversed to within a short period after the administration of a solution containing a normal concentration of potassium ions, reflecting the importance of timely electrolyte supplementation.

This study directly and clearly demonstrates the changes in conduction direction caused by low potassium. There were differences in the starting heart rate between the different experimental groups, and 6 Hz stimulation of the apical part of the isolated heart could unify the ventricular rate to 360 beats/min in each experimental group, allowing us to better compare indices between the different treatment groups. As the potassium concentration decreased, the direction of atrial, ventricular, and 6 Hz ventricular conduction could be altered, and the conduction heatmap was extremely disturbed in the period before ventricular fibrillation. Under sinus rhythm and 6 Hz stimulation, hypokalaemia had a significant inhibitory effect on atrial-ventricular conduction. Hypokalaemia significantly increased absolute atrial and ventricular dispersion under sinus rhythm, whereas under 6 Hz stimulation of the apical region, which exhibits a very high degree of autoregulation, no significant increase in absolute ventricular dispersion was observed as a result of hypokalaemia. Dispersion reflects the heterogeneity of conduction and is clearly correlated with arrhythmia. Greater dispersion results in chaotic signalling on the surface of the heart, which makes arrhythmia more likely to occur. The data showed that atrial and ventricular dispersion was significantly prolonged under sinus rhythm in the presence of low potassium concentrations but that 6 Hz stimulation had no effect on ventricular dispersion; however, we cannot rule out arrhythmia susceptibility was still high under these conditions. A significant increase in AVD under sinus rhythm caused by a decrease in the potassium ion concentration has been demonstrated in numerous clinical cases (29). Decreased conduction velocity is one of the factors leading to atrial fibrillation or ventricular fibrillation (30), and retrace is closely related to arrhythmia (31). In combination with the prolongation of the atrial-ventricular conduction time, a decrease in conduction velocity, a change in the direction of conduction, an increase in conduction dispersion, and retrace are likely involved in the occurrence of arrhythmia due to hypokalaemia.

A stimulating electrode was used to stimulate the apex of the heart to produce stable excitation, and the threshold current and ERP of cardiac excitation were measured by gradually shortening the stimulation interval. Previous studies have shown that hypokalaemia leads to prolonged myocardial refractory period, and the results obtained in the present study are consistent with the results of experiments on isolated guinea pigs hearts conducted by Osadchii et al. (32). Hypokalemia mainly causes partial depolarization of cardiac muscle cells by affecting the conductance of potassium channels. This depolarization accelerates the inactivation of sodium channels, reducing the number of available sodium channels during subsequent depolarization, thereby shortening the ERP. The repolarization process is prolonged due to the reduced potassium efflux. The prolongation of the QT interval is caused by delayed repolarization at the end (33). However, ERP is more dependent on the early repolarization and the recovery of sodium channels (34). Therefore, although the QT interval is prolonged, ERP shows a trend of shortening. This indicates that new action potentials may occur during ventricular repolarization, leading to the occurrence of early afterdepolarizations (EADs). The isolated heart model used in this study excluded neurohumoral regulation and provided a clearer view of the intrinsic electrophysiological changes. As the potassium concentration further decreases, the risk of EADs gradually increases, as indicated by gradual increases in the frequency of atrial and ventricular arrhythmia and the frequency of malignant arrhythmia as the potassium concentration decreases.

Transcriptomic analysis revealed that low-potassium treatment could lead to changes in gene expression. This study focuses on gene expression within the myocardium, reflecting the changes in the heart's own gene expression, thereby enriching the biological processes that it may affect, rather than systemic metabolic regulation. In the future, it can be combined with *in vitro* or *in vivo* models to explore the impact of fatty acid metabolism. Furthermore, the Langendorff system is widely used for direct studies on cardiac electrophysiology and drug effects, eliminating the interference from neuroendocrine factors. This study aims to reveal the direct effects of rhodanine glycoside on cardiac conduction and gene expression, laying the foundation for subsequent *in vivo* studies that combine systemic factors. The differential expression genes identified in this study were screened using strict criteria (FDR < 0.05, Fold Change > 1.5), resulting in a relatively small number of detected genes. However, some of the core differentially expressed genes still have clear biological significance. Moreover, the transcriptional regulation of the heart muscle by acute electrolyte disturbances may be more limited, relying more on rapid ion channel regulation rather than gene expression.

Among the significantly upregulated genes, Mt-nd 6 is an important factor involved in mitochondrial oxidative phosphorylation, suggesting that low-potassium treatment can affect mitochondrial energy metabolism. GO enrichment analysis revealed that low-potassium treatment could trigger positive regulation of the immune response and negative regulation of leukocyte proliferation and macrophage colony-stimulating factor signalling pathway, etc. All these processes are closely related to immune and inflammatory processes, suggesting that low potassium concentrations could have an effect on immune and inflammatory processes; however, whether low potassium concentrations exert effects through mitochondrial potassium channels still needs to be further studied. After SAL treatment, alterations in gene expression were observed, and the above changes in the expression of genes related to energy metabolism, the inflammatory response, the immune response and other related processes caused by low potassium concentrations were abolished, indicating that SAL could affect gene expression in isolated rat hearts treated with low-potassium solutions and inhibit alterations in energy metabolism, the inflammatory response, and the immune response. KEGG enrichment analysis revealed that the genes CSf 1 and Ccl 12, which are involved in the MAPK signalling pathway and cell‒cytokine interactions, respectively, were significantly downregulated in the low-potassium-treated group compared with the control group, and low-potassium-induced inflammatory and immune responses might be related to these pathways. Adipoq and plin 1, which are involved in the PPAR signalling pathway, were upregulated in the SAL-treated group, which indicated that SAL might in part exert its effects through the PPAR signalling pathway. For instance, activation of PPAR can inhibit pro-inflammatory signals such as NF-*κ*B and improve energy metabolism. These effects may indirectly contribute to stabilizing myocardial electrical activity. However, the current evidence is insufficient and needs to be further verified. Future studies need to conduct *in vitro* experiments combined with PPAR pathway-specific inhibitors or agonists/antagonists and detect key downstream molecules to directly verify whether rhodanine glycoside mediates its protective effect on hypokalemic hearts through the PPAR pathway, especially its anti-arrhythmic effect (35). In the treatment of hypokalaemia, it is indisputable that the first priority is to correct the electrolyte imbalance by replenishing potassium. SAL may be used as an adjunctive drug in the future to reduce the risk of arrhythmia associated with hypokalaemia and may protect the heart to some extent in cases of malignant arrhythmia.

In this work, isolated hearts were studied, reducing the influence of various uncontrollable factors *in vivo*. Moreover, by using isolated hearts, we were able to more intuitively analyse the influence of drugs on the heart, and experiments involving isolated hearts are stable and repeatable. There are several limitations to this study. In clinical practice, hypokalemia is usually closely associated with elevated levels of renin or aldosterone. However, the isolated hearts cannot be regulated in this way. Therefore, in the future, the overall protective effect of SAL needs to be verified through *in vivo* animal models. In addition, while we used low-potassium solutions in the experiment, we did not consider the effects of the concentration of chloride ions and differences in osmolality caused by chloride ions, which may have affected the results of the experiment to a certain extent. Previous studies have shown that low chloride may cause cellular acid-base imbalance by inhibiting the Cl^−^/HCO_3_^−^ exchanger in cardiac muscle cells, leading to an extension of the repolarization phase of the action potential and ultimately resulting in a significant prolongation of the QT interval (36). Low chloride may also further prolong the action potential duration by inhibiting the activity of the CLC-3 chloride channel and reducing chloride icons efflux during repolarization (37). Moreover, low chloride may affect the repolarization process of the action potential, thereby influencing the conduction function of the heart (38). Therefore, in future studies, it is necessary to specifically clarify the effect of chloride ions on the electrophysiology of the heart.

## 6 Conclusion

This study investigated the effects of low-potassium solutions on the heart and the role of SAL through experiments on rats. The results showed that low-potassium solutions had significant impacts on cardiac ECG signals. The ECG changes caused by low-potassium solutions were unevenly distributed throughout the cardiac cycle, involving alterations in heart rate, PR interval, QRS interval, and QT interval. Hypokalaemia inhibited cardiac conduction in a dose-dependent manner, increasing the dispersion of the left atrium and left ventricle and altering the conduction direction. SAL could significantly inhibit the increase in atrial and ventricular dispersion induced by hypokalaemia, showing a trend of improving cardiac conduction and significantly reducing the incidence of ventricular fibrillation in isolated rat hearts treated with low-potassium solutions. Moreover, low-potassium treatment interfered with the inflammatory response, immune response, and energy metabolism, while SAL could inhibit these interferences caused by low potassium.

## Data Availability

The raw data supporting the conclusions of this article will be made available by the authors, without undue reservation.
