# Supplementary material for: Salidroside ameliorates abnormalities in electrophysiological indices induced by perfusion of the heart with low-potassium solutions
Source: Front Cardiovasc Med. 2025 Aug 12;12:1628940. doi: 10.3389/fcvm.2025.1628940 (PMC12378503; doi:10.3389/fcvm.2025.1628940)
Supplement: Supplementary file 1 [file Table1.docx]

**Table S1.** Equipment

| Equipment Name |  | Manufacturer |
| --- | --- | --- |
| 64-channel calibration acquisition system |  | Mapping Lab Inc. |
| 64-channel calibration amplifier pen-like Recording electrodes |  | Mapping Lab Inc. |
| ECG Detection Module Constant Current or Voltage Stimulator |  | Mapping Lab Inc. |
| Constant temperature water bath |  | Changzhou Huaguan Instrument Manufacturing Co. |

**Table S2.** Experiment reagent

| Name of drugs | Manufacturer | Catalogue No. | Purity |
| --- | --- | --- | --- |
| NaCl | Sigma Corporation | V900058 | ≥99.0% |
| KH_2_PO_4_ | Sigma Corporation | V900041 | ≥99.0% |
| KCl | Sigma Corporation | P9541 | ≥99.0% |
| CaCl_2_-2H_2_O | Sigma Corporation | C3306 | ≥99.0% |
| NaHCO_3_ | Sigma Corporation | S5761 | ≥99.5% |
| D-glucose | Sigma Corporation | G7528 | ≥99.5% |
| MgCl_2_-6H_2_O | Sigma Corporation | 63064 | ≥99.0% |

**Table S3.** K-H solution configuration methods

| Name (of a thing) | C (mmol/L) | MW (g.mol^-1^) | g/L |
| --- | --- | --- | --- |
| NaCl | 119 | 58.44 | 6.9544 |
| NaHCO_3_ | 25 | 84.01 | 2.1003 |
| KCl | 4 | 74.55 | 0.2982 |
| KH_2_PO_4_ | 1.2 | 136.08 | 0.1633 |
| MgCl_2_-6H_2_O | 1 | 95.2 | 0.0952 |
| CaCl_2_-2H_2_O | 1.8 | 146.98 | 0.2646 |
| D-glucose | 10 | 180.16 | 1.8016 |

Note: KCl, KH_2_PO_4_, MgCl_2_ can be prepared as 10 × mother liquor, placed in the refrigerator at 4 ℃, used within 7 days; K-H solution placed in the refrigerator at 4 ℃, ready to use, used within one day.Heparin sodium was diluted to 1000 U/mL in saline, stored at -4°C in the dark, and removed when ready for use.

**Table S4.** Statistical table of potassium ion concentration in perfusion solution of each treatment group

|  | Potassium ion concentration（mmol/L） | Mean（mmol/L） |
| --- | --- | --- |
| Control groups | 4.98 | 5.38 |
|  | 5.49 |  |
|  | 5.66 |  |
| Low-potassium-treated-groups | 2.85 | 2.75 |
|  | 2.80 |  |
|  | 2.59 |  |
| Salidroside-treated-groups | 2.53 | 2.54 |
|  | 2.63 |  |
|  | 2.74 |  |
